# Hubs to spread technology and save lives

**DOI:** 10.2471/BLT.15.020515

**Published:** 2015-05-01

## Abstract

The patent on an expensive preventive treatment for respiratory syncytial virus infections expires this year. A WHO technology transfer hub in the Netherlands aims to help developing countries make the drug themselves. Gary Humphreys reports.

Every year, from November for about 18 weeks, the paediatric ward at the Kilifi District Hospital, an hour north of Mombasa on the Kenyan coast, admits more cases of severe acute respiratory infection associated with respiratory syncytial virus (RSV) infections than anything else.

While healthy children with RSV infections tend to experience mild cold-like symptoms, less robust infants – preterm or those with congenital heart disease – may face severe illness and need to be hospitalized. 

The consequences can be fatal.

“Despite the importance of RSV, it tends to be neglected – especially by policy-makers,” says Dr Charles Sande, a post-doctoral immunologist working on the immune response to RSV in children at the Centre of Geographical Medicine Research in Kilifi.

“Despite the importance of RSV, it tends to be neglected – especially by policy-makers.”

Charles Sande

His frustration is shared by Dr Louis Bont, who heads RSV research at the University Medical Centre in Utrecht in the Netherlands and chairs ReSViNET, a global consortium of RSV researchers. “RSV causes 6.7% of post-neonatal [after the first 28 days] deaths in children in the first year of life,” he says. “Only malaria causes more deaths, and yet RSV is rarely talked about as a public health priority.”

Published estimates from Kilifi show that overall RSV prevalence in children aged up to 12 months in this region is about 20%, rising to 32% during seasonal epidemics, with RSV-associated mortality of 2.2%, although there are no data on this on a national level in Kenya or globally for that matter.

**Figure:**
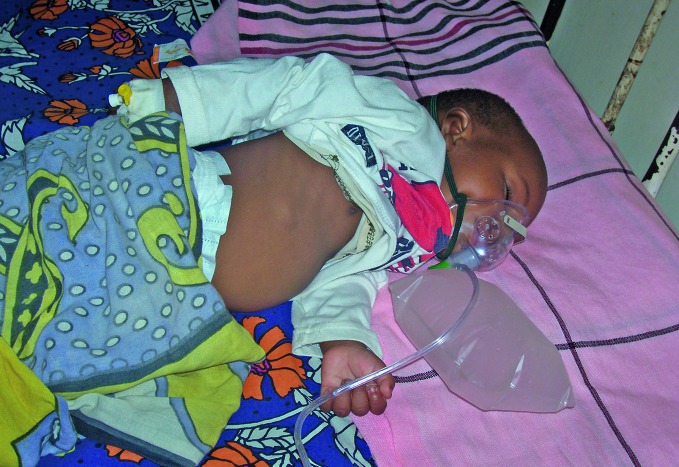
An infant with severe RSV-related pneumonia undergoing oxygen therapy in Kenya

Kemri-Wellcome Trust/Mwanajuma Ngama

A systematic review of recent surveys published in the *Lancet* in 2010 estimated that each year as many as 200 000 children younger than 5 years die from acute lower-respiratory-tract infections caused by RSV.

However fuzzy the overall epidemiological picture may be, it is clear that deaths from RSV nearly all occur in developing countries.

There is no specific treatment for RSV infections and the only preventive treatment available, palivizumab, developed and patented by MedImmune, a United States-based pharmaceutical company, and marketed under the brand name Synagis®, is expensive. A treatment course costs US$ 9615 in the United States of America, and about US$ 5380 (€5000) in Europe.

Palivizumab is indicated as a preventive treatment for children who are at high risk of severe RSV infections and has been shown to reduce RSV-related hospitalizations in pre-term infants by about 80%.

But, according to scientist Martin Friede, who leads the technology transfer team at the World Health Organization (WHO) in Geneva, a biosimilar version of palivizumab could be produced for around US$ 250 per treatment course.

After MedImmune’s palivizumab patent expires in the European Union in August and in the United States in October, countries can produce their own affordable copies of the patented drug with the help of a new WHO technology transfer hub set up in Utrecht in the Netherlands.

The new hub – established in March – aims to make patent-free biotech drugs affordable in developing countries. Its starting project – the development of biosimilar palivizumab – is in collaboration with Argentina-based biotherapeutics company, Chemo.

Biotherapeutics – also known as biotech drugs, biologics or biopharmaceuticals – are therapies derived from living organisms, rather than by chemical synthesis. A biopharmaceutical, for example penicillin, is a macromolecule or cellular component that originates from a living organism and that is used as a pharmaceutical product. Palivizumab is an antibody generated from cultures of genetically identical cells, or clones.

It is the third time that WHO has established a hub to encourage the spread of technology to address public health needs and overcome the problem of inequitable access due to high costs for developing countries.

The first was set up in 2007 at the Netherlands Vaccine Institute, a Dutch governmental vaccine manufacturer with a long history of technology transfer projects, to support the establishment of new influenza vaccine production in developing countries.

The hub brought together all the appropriate development, clinical and regulatory expertise, while generating a comprehensive documentation package (standard operating procedures, batch process records, validation procedures, etc.) and organizing the quality control and management so that products produced locally meet the required standards for obtaining marketing authorization.

A second hub, the Vaccine Formulation Laboratory, was established in 2010 at the University of Lausanne in Switzerland to provide know-how and training on adjuvant technology. Adjuvants are used in vaccine development to boost the human immune response to vaccines.

The Lausanne hub has transferred adjuvants to vaccine manufacturers in developing countries to help stretch supplies of pandemic influenza vaccines and it is currently running a new programme on adjuvants for inactivated polio vaccines, to make these vaccines more affordable for developing countries.

Friede is a keen supporter of the hub approach. “Among other things, hubs allow for efficient multilateral technology transfer as opposed to the usual, slower, country-to-country arrangements,” he says.

**Figure:**
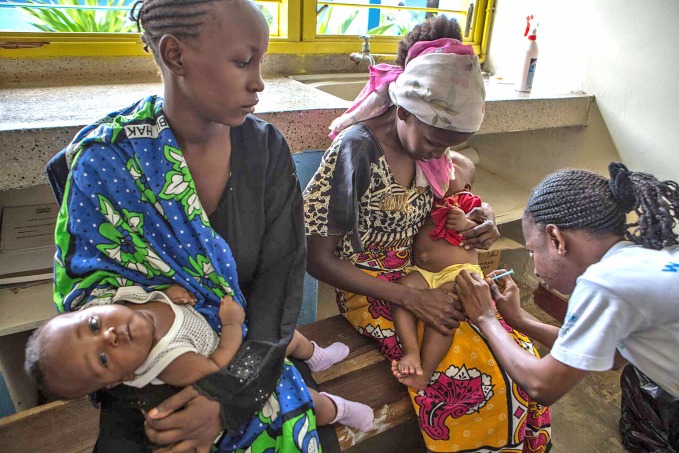
Children take part in a study of respiratory infections in Kilifi accompanied by their mothers

GAVI/Evelyne Hockstein

According to Professor Huub Schellekens of Utrecht University’s Department of Pharmaceutical Sciences and Department of Innovation Studies, one of the key collaborators in Utrecht, the hub will not only serve to spread the relevant technology and expertise, but will in fact be doing a great deal of the work needed to make biosimilar palivizumab a reality.

“The production of biosimilar palivizumab (a monoclonal antibody) is itself not rocket science,” Schellekens says, “but as a biopharmaceutical it does present a number of challenges compared with producing simpler chemically-synthesized drugs.”

Copying a biopharmaceutical is not the same as copying a chemical component or compound. Each living organism is slightly different and the resulting biosimilar product is also dependent on the manufacturing process, which can affect the way the biosimilar product performs. That is why much of the effort and expense involved in developing a biosimilar product is invested in testing how well it works and ensuring that it is safe.

Schellekens believes that the Utrecht hub will make the actual transfer of technology relatively straightforward by giving local country-level producers the clones generated at the hub, so that they can extract palivizumab themselves.

The hub will also support countries by organizing the entire pre-clinical and clinical development programme to test the clone, he says, as well as organizing the quality control and management needed to ensure that the products produced locally in developing countries meet criteria allowing the use of centrally collected pre-clinical data to obtain marketing authorization. While some agreement has been reached internationally on how biosimilars will be regulated, it is not yet clear that all countries will adopt the same approach for authorization.

“By establishing standardized production and purification methods, it will be possible for countries to locally produce palivizumab that is highly similar to the biosimilar used in the clinical studies,” he says.

The resulting palivizumab will, in fact, be so close to the original that countries producing it may not need to test it in large clinical trials.

They will, however, need to conduct bridging studies to demonstrate comparability to the palivizumab clone produced by the hub, but these studies will be less onerous than full-blown trials.

Says Schellekens: “If each country had to do their own clinical trials with material that they produced locally, it would cost around US$ 50 million per product per producer. With this system we can avoid those costs and bring down the final cost of a treatment course to around US$ 250 and maybe even less.”

“We want to make participation as fair as possible,” says Schellekens, who recognizes nevertheless that countries altogether lacking in production capacity may be sidelined in the process. So wouldn’t it make more sense to allow those countries with the industrial capacity to produce biosimilar palivizumab for everyone? Schellekens doesn’t think so.

“We want as many countries as possible to be autonomous, and technology transfer is the best way to achieve that.”

Huub Schellekens

“A number of companies have approached us saying that they’re ready to make this for the whole world, and we’ve said that while that might solve the short-term medical problem it is not a long-term solution. We want as many countries as possible to be autonomous, and technology transfer is the best way to achieve that.”

